# Cardiovascular magnetic resonance imaging and spectroscopy in clinical long-COVID-19 syndrome: a prospective case–control study

**DOI:** 10.1186/s12968-022-00887-9

**Published:** 2022-09-12

**Authors:** Miroslawa Gorecka, Nicholas Jex, Sharmaine Thirunavukarasu, Amrit Chowdhary, Joanna Corrado, Jennifer Davison, Rachel Tarrant, Ana-Maria Poenar, Noor Sharrack, Amy Parkin, Manoj Sivan, Peter P. Swoboda, Hui Xue, Vassilios Vassiliou, Peter Kellman, Sven Plein, Stephen J. Halpin, Alexander D. Simms, John P. Greenwood, Eylem Levelt

**Affiliations:** 1 Multidisciplinary Cardiovascular Research Centre and Biomedical Imaging Science Department, Leeds Institute of Cardiovascular and Metabolic Medicine, University of Leeds, Leeds, LS2 9JT UK; 2 Department of Rehabilitation Medicine, Leeds Teaching Hospitals Trust, Leeds, UK; 3 Leeds Community Healthcare NHS Trust, Leeds, UK; 4 Department of Rehabilitation Medicine, Leeds Institute of Rheumatic and Musculoskeletal Medicine, University of Leeds, Leeds, UK; 5 National Heart, Lung, and Blood Institute, National Institutes of Health, DHHS, 10 Center Drive MSC-1061, Bethesda, MD 20892 USA; 6 Department of Cardiovascular and Metabolic Health, University of East Anglia, Norwich, UK; 7 Department of Cardiology, Leeds Teaching Hospitals Trust, Leeds, UK

**Keywords:** COVID-19, Post-COVID-19 syndrome, LONG COVID, Cardiovascular magnetic resonance imaging, 31-phosphorus magnetic resonance spectroscopy

## Abstract

**Background:**

The underlying pathophysiology of post-coronavirus disease 2019 (long-COVID-19) syndrome remains unknown, but increased cardiometabolic demand and state of mitochondrial dysfunction have emerged as candidate mechanisms. Cardiovascular magnetic resonance (CMR) provides insight into pathophysiological mechanisms underlying cardiovascular disease and 31-phosphorus CMR spectroscopy (^31^P-CMRS) allows non-invasive assessment of the myocardial energetic state. The main aim of the study was to assess whether long COVID-19 syndrome is associated with abnormalities of myocardial structure, function, perfusion and energy metabolism.

**Methods:**

Prospective case–control study. A total of 20 patients with a clinical diagnosis of long COVID-19 syndrome (seropositive) and no prior underlying cardiovascular disease (CVD) and 10 matching healthy controls underwent ^31^P-CMRS and CMR at 3T at a single time point. All patients had been symptomatic with acute COVID-19, but none required hospital admission.

**Results:**

Between the long COVID-19 syndrome patients and matched contemporary healthy controls there were no differences in myocardial energetics (phosphocreatine to ATP ratio), in cardiac structure (biventricular volumes), function (biventricular ejection fractions, global longitudinal strain), tissue characterization (T_1_ mapping and late gadolinium enhancement) or perfusion (myocardial rest and stress blood flow, myocardial perfusion reserve). One patient with long COVID-19 syndrome showed subepicardial hyperenhancement on late gadolinium enhancement imaging compatible with prior myocarditis, but no accompanying abnormality in cardiac size, function, perfusion, extracellular volume fraction, native T1, T2 or cardiac energetics.

**Conclusions:**

In this prospective case–control study, the overwhelming majority of patients with a clinical long COVID-19 syndrome with no prior CVD did not exhibit any abnormalities in myocardial energetics, structure, function, blood flow or tissue characteristics.

## Background

While initial public health responses focused on reducing the acute burden of coronavirus disease 2019 (COVID-19), a growing body of evidence indicates that severe acute respiratory syndrome coronavirus 2 (SARS-CoV-2) infection can also result in long-term multisystem sequelae even after a mild acute SARS-CoV-2 infection [1]. The current definition of the Post-COVID-19 syndrome includes persistent symptoms and/or long-term complications of SARS-CoV-2 infection beyond 12 weeks from the onset of the infection [2]. The global impact of long COVID-19 syndrome, with the high burden of self-reported symptoms, impaired quality of life, limitations in exercise tolerance and cognitive function, has been profound. In non-hospitalised patients with mostly mild symptoms during the acute SARS-CoV-2 infection, the long COVID-19 syndrome affects up to 27.8% of adults [3, 4].

Persistent symptoms suggestive of cardiovascular involvement are common even in previously healthy and non-hospitalised COVID-19 patients. These include fatigue (63–98%), breathlessness (37–70%), chest pain (16–60%) and palpitations (16%) [5]. It has been suggested that the long-term sequelae in long COVID-19 syndrome patients may include increased cardiometabolic demand and a state of mitochondrial dysfunction as a result of oxidative stress triggered by the viral infection [2].

Cardiovascular magnetic resonance (CMR) allows comprehensive evaluation of myocardial structure, function, strain, tissue characteristics, fibrosis and perfusion with excellent reproducibility [6]. Cardiac 31-phosphorus CMR spectroscopy (^31^P-CMRS) allows for the measuring of the relative concentration of phosphocreatine (PCr) to adenosine triphosphate (ATP) (PCr/ATP) in the heart which is a marker of the myocardium’s ability to convert substrate into ATP for active processes, a sensitive index of the energetic state and the cardiometabolic status of the heart [7].

As there is only scarce data available from prospective CMR studies in non-hospitalised, previously healthy individuals with long COVID-19 syndrome it is currently unknown whether it is associated with abnormalities of myocardial structure, function, perfusion and tissue characteristics or energetic derangement. Combining CMR and ^31^P-CMRS in an observational prospective case–control study we sought to assess cardiac involvement in long COVID-19 syndrome in previously healthy and non-hospitalised patients.

## Research design and methods

This single-center cross-sectional study complied with the Declaration of Helsinki. It was approved by the Research Ethics Committee (REC18/YH/0168) and informed written consent was obtained from each participant. The data that support the findings of this study are available from the corresponding author on reasonable request.

### Participants

**Fig. 1:**
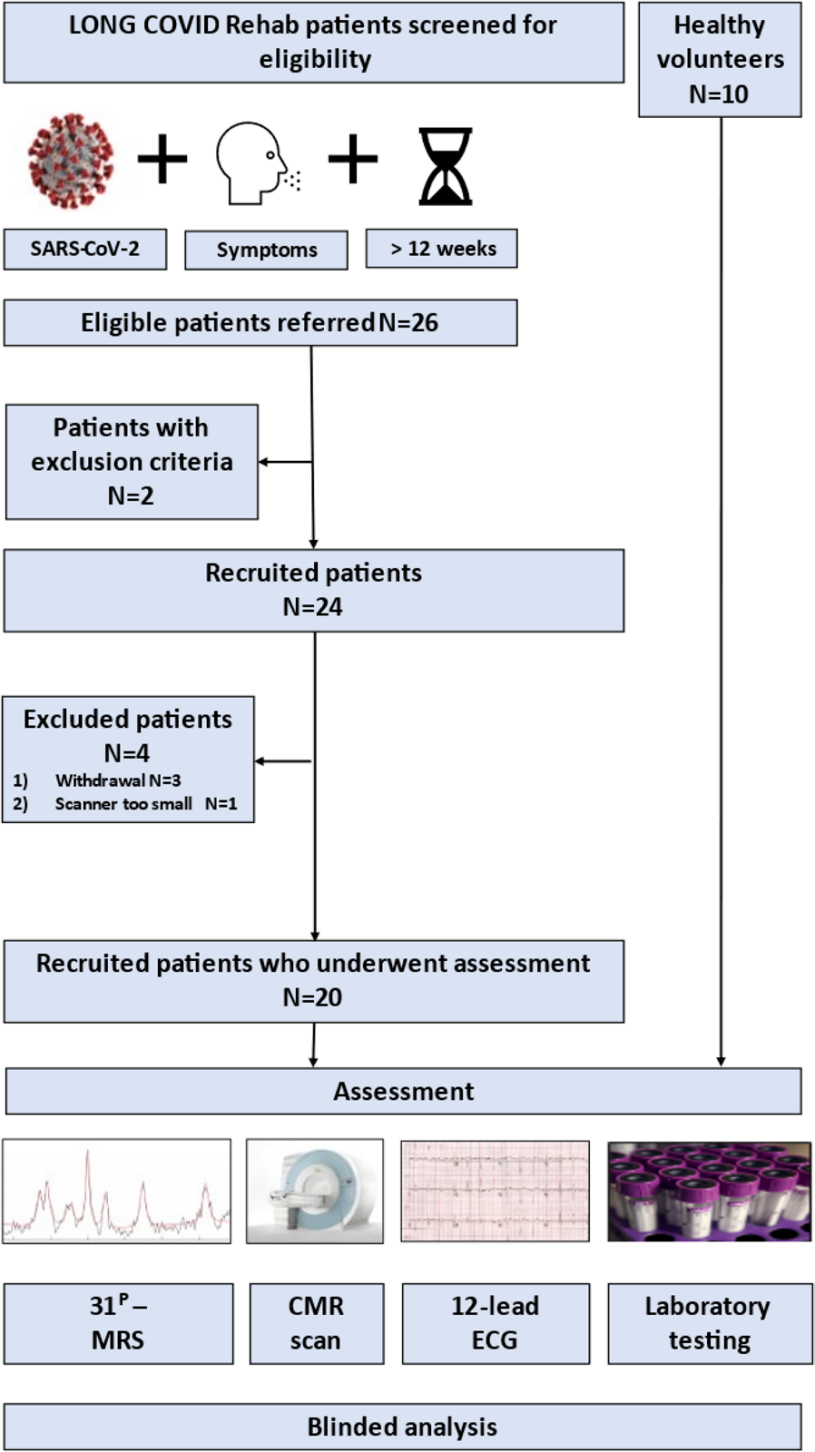
Study flow chart of participant recruitment

Twenty participants at least 12 weeks after a laboratory-confirmed (SARS-CoV-2 polymerase chain reaction positive) acute SARS-CoV-2 infection with persistent symptoms and a clinical diagnosis of long COVID-19 syndrome were prospectively recruited between March 2021 and July 2021 from the Leeds Teaching Hospitals NHS Trust (LTHT) LONG COVID Rehabilitation Clinic. Patients were approached by their medical team at the time of clinical review and invited to participate in our study. Ten healthy subjects without a previous COVID-19 diagnosis and of similar age and sex distribution formed the healthy control group. A flow chart of participant recruitment is shown in Fig. 1.

#### Inclusion and exclusion criteria

Long COVID-19 syndrome was diagnosed by multidisciplinary assessment in the LONG COVID Rehabilitation Clinic according to the UK NICE guidelines: (1) signs and symptoms that have developed during or after a presumed or confirmed COVID-19 infection (all patients included were seropositive); (2) continued symptoms for more than 12 weeks; and (3) alternative diagnoses have been excluded. Patients had to have ongoing symptoms at the time of assessment [8].

Patients with known coronary artery disease, cerebrovascular disease, cardiac surgery, atrial fibrillation, moderate or above valvular heart disease, hypertension, any type of diabetes, renal impairment, chronic pulmonary obstructive disease, resolution of symptoms at the time of assessment, and participants with contraindications to CMR were excluded.

Healthy subjects had no symptoms, no prior COVID-19 diagnosis, no past medical history of cardiovascular or respiratory disease and no history of hypertension or any type of diabetes.

### Study protocol

Patients and healthy subjects underwent identical assessments and ^31^P-CMRS/CMR imaging protocols.

#### Clinical data

Details on clinical symptoms, signs, laboratory findings at the LTHT LONG COVID Rehabilitation Clinic were extracted from electronic medical records. Impact of symptoms on quality of life and activities of daily living was assessed by means of the EQ-5D-5L questionnaire. Patient medication history, blood test results and chest radiographic imaging were recorded (where applicable). Symptom severity was categorized using self-reported COVID-19 Yorkshire Rehabilitation Screening questionnaire performed at the time of the assessment in the rehabilitation clinic.

#### Anthropometric measurements

During the single visit to the research center, height and weight were recorded, body mass index (BMI) was calculated, blood pressure (BP) was recorded (DINAMAP-1846-SX, Critikon Corporation, General Electric Healthcare, Chicago, Illinois, USA). A blood sample was taken from each participant for assessment of full blood count, estimated glomerular filtration rate (eGFR) and N-terminal pro hormone B-type natriuretic peptide (NT-proBNP) levels.

#### ^31^Phosphorus-cardiovascular magnetic resonance spectroscopy (^31^P-CMRS)

^31^P-CMRS was performed to obtain the PCr/ATP from a voxel placed in the mid-ventricular septum, with subjects lying supine and a ^31^P transmitter/receiver cardiac coil (Rapid Biomedical GmbH, Rimpar, Germany) placed over the heart, in the iso-center of the magnet on a 3T CMR system (Prisma, Siemens Healthineers, Erlangen, Germany) as previously described [9].

#### Cardiovascular magnetic resonance (CMR)

**Fig. 2:**
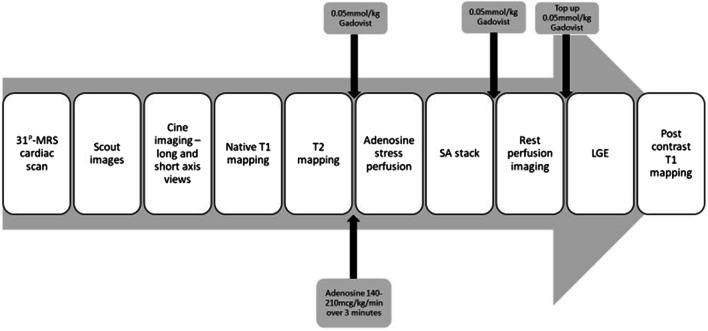
Study CMR protocol. Multi-parametric cardiovascular magnetic resonance included ^31^P-CMR spectroscopy (CMRS) (20 min). This was followed by CMR, which included cine imaging to assess left ventricular (LV) volumes, mass and ejection fraction and strain parameters; native pre-contrast and native post contrast T1 mapping for measuring T1 values and extracellular volume fraction; adenosine stress perfusion imaging for assessment of myocardial rest and stress blood flow and myocardial perfusion reserve; late gadolinium enhancement (LGE) imaging for measuring myocardial scar percentage

^31^P-CMRS study was followed by CMR using the same scanner after a coil change. The CMR protocol (Fig. 2) consisted of cine imaging using a balanced steady-state free precession (bSSFP) sequence, native pre- and post-contrast T1 mapping, stress and rest perfusion and late gadolinium enhancement (LGE).

Native T1 maps were acquired in 3 short-axis slices, including segments with maximal wall thickness, using a breath-held modified look-locker inversion recovery (MOLLI) acquisition as previously described [10]. Post-contrast T1 mapping acquisition was performed 15 min after last contrast injection.

Extracellular-volume fraction (ECV) was estimated based on the native and post contrast septal myocardial and blood T1 values and haematocrit (obtained from full blood count sample taken on the day of assessment). Dynamic steady state between the plasma and the interstitium was achieved by acquisition of the second T1 map 15 min after gadolinium administration. ECV was therefore quantified as change of concentration of gadolinium in the myocardium relative to the concentration in blood in this dynamic steady state, according to the equation: ECV = (1 − haematocrit)*[(1/post contrast myocardial T1 − 1/native myocardial T1)/(1/post contrast blood T1-1/native blood T1)] as previously described [11].

T2 maps were acquired in 3 short-axis slices using a T2 prepared true fast imaging with balanced steady state free precession (bSSFP) pulse sequence to produce single‐shot T2 prepared images, each with different T2 preparation times as previously described [12]. The T2 prepared bSSFP images were acquired with 3 recovery heartbeats to allow for sufficient magnetization recovery in between acquisitions [13]. T2 was estimated by pixel‐wise fitting assuming monoexponential signal decay, and a color, scaled, motion‐corrected myocardial T2 map was then generated [13].

Perfusion imaging used free-breathing, motion-corrected automated in-line perfusion mapping [14]. Participants were advised to avoid caffeine for 24 h before the study. For stress perfusion imaging, adenosine was infused at a rate of 140 µg/kg/min and increased up to a maximum of 210 µg/kg/min according to haemodynamic and symptomatic response (a significant haemodynamic response to adenosine stress was defined as > 10 beats/min increase in heart rate, or BP drop > 10 mmHg and > 1 adenosine-related symptom e.g., chest tightness, breathlessness) [15]. For perfusion imaging, an intravenous bolus of 0.05 mmol/kg gadobutrol (Gadovist, Leverkusen, Germany) was administered at 5 ml/s followed by a 20 ml saline flush using an automated injection pump (Medrad MRXperion Injection System, Bayer Healthcare, Berlin, Germany). Perfusion mapping was performed and implemented on the scanner using the Gadgetron streaming software image reconstruction framework as previously described [14]. Short axis cine acquisition was performed for biventricular volume and function assessments after a minimum of 5 min delay following the discontinuation of adenosine, to allow sufficient washout of the vasodilator and for the heart rate to return to pre-adenosine administration resting values.

Late gadolinium enhancement (LGE) CMR was performed using a phase-sensitive inversion recovery (PSIR) sequence in the left ventricular (LV) short-axis planes, and long axis planes > 8 min after gadolinium administration [16].

#### Quantitative analysis

All ^31^P-CMRS and CMR post-processing analyses were performed off-line blinded to all participant details by ST and NJ after completion of the study. The anonymization codes were only unlocked once all data analysis was completed.

All CMR image analysis was performed by MG using cvi42 software (Circle Cardiovascular Imaging, Calgary, Canada) and reviewed by EL. Images for biventricular volumes and function were analysed as previously described [17].

Global longitudinal shortening (GLS) data were derived from horizontal long-axis and vertical long-axis images, and image reconstruction and processing were implemented using the Gadgetron software framework with the previously developed convolutional neural network for labelling landmarks on CMR images. The performance of this network was shown to be comparable to manual labeling [18].

Myocardial perfusion image reconstruction and processing was implemented using the Gadgetron software framework as previously described [14]. Rest/stress myocardial blood flow (MBF) were measured for each of the 16-segments using the American Heart Association (AHA) classification. MBF values for 16-segments were averaged to provide a global value.

Native T1 and T2 maps were analyzed using cvi42 software (Circle Cardiovascular Imaging) which were measured for each of the 16-segments using the AHA classification as previously described [13, 19].

LGE images were analysed qualitatively as either LGE present or absent. If present, the location was described as per AHA 16-segment model and the myocardial distribution pattern described.

#### Definition of cardiac injury

Study definitions for both myocardial and pericardial involvement with imaging components were adapted from the Updated Lake Louise Imaging Criteria [20] as previously described by Moulson and colleagues [21].

### Statistical analysis

Statistical analysis was performed using Minitab (version 19, Minitab, LLC, State College, Pennsylvania, USA). Data were examined for normality using the Shapiro–Wilk test. Normally distributed variables were expressed as mean ± standard deviation; non-normal as median (interquartile range). Proportions were expressed as percentages. Comparisons of all ^31^P-CMRS, CMR and biochemistry data between patients and healthy controls were performed with 2-sample t-test or Mann–Whitney as appropriate. Categorical variables were compared with Pearson’s chi-square test. A priori sample size calculation was performed to detect a 10% difference in PCr/ATP ratio between the controls and participants with long COVID-19 syndrome. Assuming two-tailed independent *t*-test analysis (with 80% power at α = 0.05) pilot data (PCr/ATP in normal populations 2.10 ± 0.25) suggested that 16 subjects would be needed for each group. P ≤ 0.05 was considered statistically significant.

## Results

### Participant demographics, biochemical and clinical characteristics

Demographics, laboratory data, symptoms and haemodynamics are shown in Table 1.

**Table 1:** Comparison of baseline characteristics between patients with long COVID-19 syndrome and healthy subjects

Variable	Healthy subjects (n = 10)	Long COVID-19 syndrome (n = 19)*	p-value
Age (years)	51 ± 11	45 ± 13	0.20
BMI (kg/m^2^)	25 ± 3	26 ± 4	0.42
Male, n (%)	6 (60)	9 (47)	0.52
Duration of symptoms at time of assessment (days)	n/a	163 [142,185]	n/a
Heart rate (beats per minute)	61 ± 6	68 ± 12	0.17
Systolic blood pressure (mmHg)	126 ± 27	119 ± 17	0.49
Diastolic blood pressure (mmHg)	70 ± 10	74 ± 9	0.30
Laboratory findings			
C-Reactive Protein at diagnosis (mg/L)	n/a	141 ± 11	n/a
White Blood Cell count at study visit (10^9^/L)	6.4 [4.8–8.9]	7.2 [5.9–8.6]	0.36
Lymphocyte count at study visit (10^9^/L)	2.0 ± 0.8	1.0 ± 2.5	0.28
Neutrophil count at study visit (10^9^/L)	3.6 [2.8–5.2]	4.4 [3.2–5.0]	0.35
Monocyte count at study visit (10^9^/L)	0.39 ± 0.15	0.46 ± 0.16	0.30
Platelet count at study visit (10^9^/L)	226 ± 54	260 ± 57	0.14
Haemoglobin at study visit (g/L)	145 [139–156]	147 [130–150]	0.71
Creatinine at study visit (µmol/L)	66 ± 14	68 ± 17	0.69
eGFR at study visit (ml/min/1.73m^2^)	90 [86–90]	90 [82–90]	0.31
NT-proBNP at study visit (ng/L)	35 [35–71]	56 [38–68]	0.24
Chest X-ray findings at index diagnosis			
Normal, n (%)	n/a	2(11)	n/a
COVID-19 changes, n (%)	n/a	3(16)	n/a
Chest X-ray findings at ≥ 12 weeks			
Normal, n (%)	n/a	4(21)	n/a
COVID-19 changes, n (%)	n/a	0(0)	n/a
Cardiovascular symptoms			
Fatigue, n (%)	n/a	16 (84)	n/a
Chest pain, n (%)	n/a	2 (11)	n/a
Dyspnoea, n (%)	n/a	10 (53)	n/a
Palpitations, n (%)	n/a	13 (69)	n/a

Continuous variables are expressed as mean [95% confidence interval], mean (SD) or median [IQR] and categorical variables as number (%). BMI: Body mass index; n: number; eGFR: estimated glomerular filtration rate; NT-pro BNP: N-terminal pro hormone B-type natriuretic peptide

**Fig. 3:**
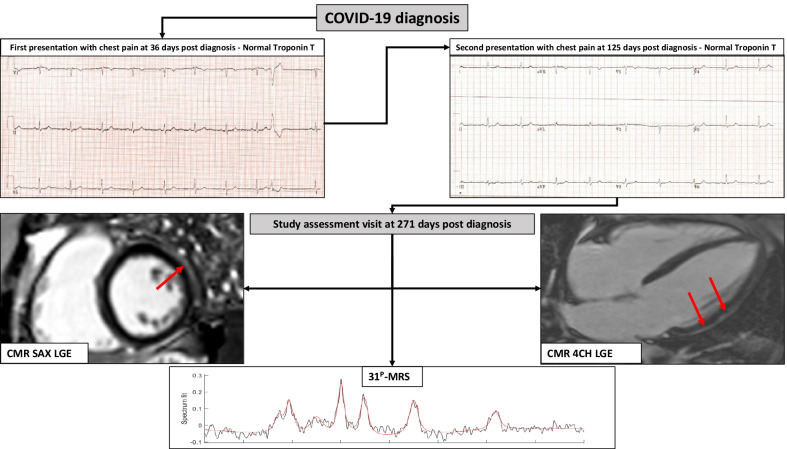
Timeline and investigations undertaken in the long COVID-19 patient with evidence of myocarditis on CMR. This patient presented first to the emergency department 36 days after diagnosis of COVID-19 with chest pain. 12-lead electrocardiogram (ECG) and cardiac biomarkers were all normal. On second presentation to the emergency department at 125 days post diagnosis, ECG and cardiac biomarkers were again normal. CMR during the study visit remonstrated evidence of prior myocarditis with subepicardial late gadolinium hyperenhancement (LGE) in the lateral wall at basal to mid-ventricular level (red arrows) in the short-axis (SAx) view (left) and 4 chamber (4Ch) view (right). 31^P^-CMRS demonstrated PCR/ATP ratio of 1.87. Other parameters were as follows: LV end-diastolic volume (LVEDV) 146 ml, LV ejection fraction (LVEF) 64%, right ventricular (RV) end diastolic volume (RVEDV) 151 ml, RV ejection fraction (RVEF) 68%, native T1 1221 ms, extracellular volume fraction (ECV) 21%, T2 43 ms, global longitudinal strain (GLS) -11.3 and myocardial perfusion reserve (MPR) 3.9

^*^1 patient with possible myocarditis scar has been excluded from the analysis and their findings presented separately in the figure (Fig. 3) legend

Of the 26 patients with long COVID-19 syndrome screened from the local LONG COVID Rehabilitation clinic, with no prior comorbidities, who had a mild course of illness during the acute phase of SARS-CoV-2 infection, 20 were recruited prospectively. There were no significant differences in age, sex or BMI between the healthy controls and the long COVID-19 group. There were also no significant differences in BP or resting heart rates between the two groups.

The numeric elevation in NT-proBNP values in the long COVID-19 group did not reach statistical significance (Healthy Controls: 35 ng/L [35–71] vs Long COVID-19 group: 55 ng/L [36–68], p = 0.24). There were no significant differences in inflammatory markers from the full blood count assessment (white cell, neutrophil and monocyte counts) or in renal profile between the groups.

Any other clinical investigations including lung imaging were undertaken at the discretion of the LTHT Long Covid Rehabilitation Clinic as per clinical indications. Only 4 patients required a repeat chest X ray imaging at 12 weeks post diagnosis, which was normal in all these cases (n = 4, 21%). One patient required a computed tomography imaging of the thorax (n = 1, 5%) and two patients were assessed with transthoracic echocardiography (n = 2, 11%). These investigations did not demonstrate any pathology.

The majority of patients received the first dose of a COVID-19 vaccine prior to CMR and ^31^P-CMRS assessments (n = 17, 85%).

### Clinical symptoms and questionnaires

Mean duration of symptoms at the time of study assessment was 163 [142,185] days. The most common cardiovascular symptoms were: fatigue (n = 16, 84%), palpitations (n = 14, 69%) and dyspnea (n = 10, 53%). A minority of patients experienced chest pain (n = 2, 11%).

Impact of symptoms on quality of life and activities of daily living was assessed by means of the EQ-5D-5L questionnaire (Table 2.) This revealed that almost 50% of long COVID-19 patients had at least moderate problems in walking about. Although only a minority of long COVID-19 patients expressed difficulty in washing and dressing themselves (n = 3; 19%), 75% of patients (n = 12) had either severe difficulty or were unable to carry out their usual daily activities such as work, studying, housework or leisure activities. A large proportion of long COVID-19 patients experienced either pain or discomfort (moderate, n = 7; 44%, severe, n = 2; 13%; extreme, n = 1; 6%). Nearly all long COVID-19 patients in our cohort experienced anxiety or depression, with over 50% of long COVID-19 patients describing these symptoms as at least moderate. Mean ‘health today’ score was 45 ± 17 reflecting poor overall health status.

**Table 2:** Results of the EQ-5D-5L questionnaire

Variable	Long COVID-19 syndrome (n = 16)
Mobility	
I have no problems in walking about	5 (31)
I have slight problems in walking about	3 (19)
I have moderate problems in walking about	7 (44)
I have severe problems in walking about	1 (6)
I am unable to walk about	0 (0)
Self-care	
I have no problems washing/dressing myself	13 (81)
I have slight problems washing/dressing myself	3 (19)
I have moderate problems washing/dressing myself	0 (0)
I have severe problems washing/dressing myself	0 (0)
I am unable to wash/dress myself	0 (0)
Usual activities	
I have no problems doing my usual activities	0 (0)
I have slight problems doing my usual activities	0 (0)
I have moderate problems doing my usual activities	4 (25)
I have severe problems doing my usual activities	9 (56)
I am unable to do my usual activities	3 (19)
Pain/discomfort	
I have no pain or discomfort	2 (13)
I have slight pain or discomfort	4 (25)
I have moderate pain or discomfort	7 (44)
I have severe pain or discomfort	2 (13)
I have extreme pain or discomfort	1 (6.3)
Anxiety/depression	
I am not anxious or depressed	1 (6)
I am slightly anxious or depressed	4 (25)
I am moderately anxious or depressed	4 (25)
I am severely anxious or depressed	4 (25)
I am extremely anxious or depressed	2 (13)
‘Health today’ score	45 ± 17

Number of patients in each category is expressed as n (%). ‘Health today’ score is expressed as mean ± SD. Scale 0–100, where 0 is the worst health imaginable, whereas 100 is the best health imaginable

### Myocardial energetics

There were no significant differences in myocardial PCr/ATP ratio between the long COVID-19 syndrome patients and the healthy controls (Healthy Controls: 2.1 ± 0.5, Long COVID-19 syndrome: 2.2 ± 0.4; p = 0.49).

### Myocardial structure and function comparisons

CMR results for biventricular volumes, systolic function and strain parameters are summarized in Table 3. The two groups were comparable in terms of LV volumes and ejection fraction (LVEF). There were no significant differences in circumferential strain, GLS or diastolic strain rates between the two groups. Right ventricular (RV) volumes and function were also comparable between the groups.

**Table 3:** Comparison of ^31^P-CMRS and CMR findings between patients with long COVID-19 syndrome and healthy subjects

Variable	Healthy subjects (n = 10)	Long COVID-19 syndrome (n = 19)	p-value
PCr/ATP ratio	2.1 ± 0.5	2.2 ± 0.4	0.49
LV end diastolic volume (ml)	158 ± 39	152 ± 22	0.68
LV end diastolic volume index (ml/m^2^)	87 ± 20	81 ± 10	0.43
LV end systolic volume (ml)	57 ± 12	60 ± 12	0.50
LV end systolic volume index (ml/m^2^)	31 ± 7	32 ± 6	0.83
LV stroke volume (ml)	93 [79–121]	87 [81–110]	0.26
LV ejection fraction (%)	64 ± 4	61 ± 4	0.07
RV end diastolic volume (ml)	170 ± 46	156 ± 29	0.41
RV end diastolic volume index (ml/m^2^)	93 ± 23	83 ± 13	0.24
RV end systolic volume (ml)	76 ± 25	67 ± 18	0.34
RV end systolic volume index (ml/m^2^)	42 ± 12	36 ± 9	0.20
RV stroke volume (ml)	93 ± 29	89 ± 17	0.64
RV ejection fraction (%)	55 ± 8	57 ± 6	0.49
Peak circumferential strain (%)	− 21.0 ± 2.1	− 20.7 ± 3.3	0.77
Global longitudinal strain (%)	− 13.3 ± 2.3	− 11.9 ± 3.7	0.21
Peak diastolic circumferential strain rate (1/s)	1.3 ± 0.2	1.3 ± 0.3	0.80
Peak diastolic longitudinal strain rate (1/s)	1.0 ± 0.2	1.0 ± 0.4	0.98
Mean T1 (ms)	1206 ± 64	1158 ± 114	0.15
Extra-cellular volume (%)	25 ± 2	22 ± 5	0.03
T2 (ms)	39 ± 2	40 ± 3	0.46
MBF rest (ml/g/min)	0.7 ± 0.1	0.8 ± 0.3	0.20
MBF stress (ml/g/min)	2.0 ± 0.5	2.1 ± 0.5	0.74
MPR	3.1 ± 0.9	3.0 ± 0.8	0.89

Continuous variables are expressed as mean (SD) or median [IQR] and categorical variables as number (%). PCr/ATP: phosphocreatine and adenosine triphosphate ratio; LV: left ventricular; ml: milliliter; ml/m^2^: milliliters per square meter of body surface area; g: grams; RV: right ventricular; MBF: myocardial blood flow; ms: milliseconds; MPR: myocardial perfusion reserve

### Myocardial tissue characteristics

There were no significant differences in global myocardial T1 (Healthy Controls: 1206 ± 64 ms, Long COVID-19 syndrome: 1158 ± 114 ms; p = 0.15) or T2 measurements (Healthy Controls: 39 ± 2 ms, Long COVID-19 syndrome: 40 ± 3 ms; p = 0.46). Although there was a statistically significant difference in the ECV between the 2 groups, the ECV was normal in both groups (Healthy Controls: 25 ± 2%, Long COVID-19 syndrome: 22 ± 5%; p = 0.03).

### Myocardial perfusion

There were no significant differences in global rest MBF (Healthy Controls: 0.7 ± 0.1 ml/min/g, Long COVID-19 syndrome group: 0.8 ± 0.3 ml/min/g; p = 0.20) or stress MBF (Healthy Controls: 2.0 ± 0.5 ml/min/g, Long COVID-19 syndrome: 2.1 ± 0.5 ml/min/g; p = 0.74). The two groups’ means of myocardial perfusion reserve were also comparable.

### Myocardial fibrosis

Hyperenhancement confined to the RV insertion point on LGE was noted in 4 long COVID-19 patients (20%) and 2 controls (20%). The global myocardial native T1 and T2 were normal in all participants. No other areas of LGE were detected in the study participants except for one long COVID-19 syndrome patient.

Of the long COVID-19 patients with a persistent symptom of chest pain, only one patient (55 years old, female, symptom duration of 271 days) was found to have previously undiagnosed isolated subepicardial pattern of scar on the CMR LGE imaging, involving the basal and mid inferolateral segments, with no associated structural or functional abnormality and normal native T1 and T2 measurements, suggestive of a possible injury reminiscent of previous myocarditis but no active inflammation. The CMR, ^31^P-CMRS and clinical data of this isolated case were excluded from all statistical analyses (Fig. 3).

## Discussion

Although the number of patients affected by long COVID-19 syndrome is continuously increasing, the underlying pathophysiology remains unknown [22]. Whilst it appears to be a multiorgan disease cardiovascular complaints are particularly common in patients with long COVID-19 syndrome diagnosis [23, 24]. This study has comprehensively evaluated this issue, by assessing myocardial energetic status, function, perfusion and tissue characterization in patients suffering from long COVID-19 syndrome. Moreover, in this study we have focused on patients who developed persistent debilitating symptoms despite a mild acute phase of the infection and no pre-existing cardiovascular disease. The results were compared with data from contemporary healthy subjects with no prior diagnosis of COVID-19.

The main findings of our study were: (1) In the overwhelming majority of the patients (19 of the 20 patients) there was no evidence of cardiac injury with no significant differences in cardiac structural and functional assessments, strain, perfusion or advanced tissue characteristics between the patients with long COVID-19 syndrome and the healthy controls; (2) There was no evidence of impaired myocardial energetic status in patients with long COVID-19 syndrome with comparable myocardial PCr/ATP ratio between the patients and the controls.

Only one patient with a symptom of chest pain showed subepicardial scar on LGE imaging suggestive of previous myocarditis, but there was no accompanying myocardial oedema, adverse remodelling, or regional/global functional abnormalities. LGE confined to the RV insertion point, which is sometimes seen in healthy populations, was detected in the same proportion of the long COVID-19 syndrome group as in the healthy control group. Consequently, our data indicate low prevalence of cardiovascular involvement with reassuring CMR findings in patients with a mild acute phase of SARS-CoV-2 infection, but persistent symptoms associated with long COVID-19 syndrome. None of the participants showed evidence of an active myocarditis-pattern injury based on Updated Lake Louise Imaging Criteria [20]. However, the dissociation between the burden of self-reported symptoms and objective measures of cardiovascular health warrants further investigation into the long-term effects of COVID-19 beyond the cardiovascular system.

There are currently no reports from other studies of patients with a clinical diagnosis of long COVID-19 syndrome following a mild acute phase of SARS-CoV-2 infection and no prior cardiovascular comorbidities. Though distinct in their study design, aims and participant characteristics, two other studies have also explored non-acute (medium or late: 2–3 months [1] and 6 months [25] from the index infection respectively) prevalence and extent of the cardiovascular sequelae after an acute SARS-COV-2 infection utilising CMR [1, 25]. Joy and colleagues have compared seropositive subjects after a mild nonhospitalized SARS-CoV-2 infection to seronegative age-, sex- and comorbidity-matched participants [25]. Six months from the index infection, at the time of CMR scanning, 11% of their total study population (seropositive and seronegative subjects) had reported on-going symptoms with no difference between the two groups. They detected no persistent cardiovascular abnormalities on CMR 6 months post–mild infection with SARS-CoV-2 compared with matched subjects [25]. Raman and colleagues have investigated survivors of a moderate to severe acute phase of COVID-19 infection 2–3 months from disease-onset at median interval of 2.3 months (IQR 2.1–2.5) and median interval of 1.6 months from discharge (IQR 1.4–1.8). They detected significantly elevated native T1 on CMR in 26% of convalescing patients but no significant difference in cardiac function or native T2 values between the patients and healthy controls [1]. Recent study of COVID-19 survivors evaluated their participants with CMR and computed tomography. The authors reflected that cardiac abnormalities found in these patients were more likely the result of pre-existing conditions, rather than COVID-19 infection. Although, not specifically focused on long COVID-19 syndrome, these findings also confirm lack of significant cardiovascular complications in this illness [26].

### Potential role of mitochondrial dysfunction in Post-COVID-19 syndrome

The mitochondria are the principal generators of cellular energy as ATP. Organ involvement in the vast majority of mitochondrial diseases is multi-systemic with a predilection for the high-energy demanding tissues [27]. These tissues depend on maintaining efficient energetic status and in times of metabolic stress, patients’ symptoms characteristically decompensate and regress [27]. The heart has a very high energy demand, while having minimal energy storing capacity [28]. Given the similarities with the clinical manifestations of mitochondrial diseases associated with genetic mutations and the symptoms of long COVID-19 syndrome including fatigue, muscle weakness, and cognitive decline along with decreased energy patterns, mitochondrial dysfunction has emerged as a candidate pathophysiological mechanism. However, no prior studies have assessed energy metabolism in patients with long COVID-19 syndrome.

^31^P-CMRS has become increasingly important in biomedical research because of its ability to measure in vivo biochemical information non-invasively [29, 30]. The energy deficient state in the heart can be detected non-invasively by ^31^P-CMRS. The relative concentration of PCr/ATP is a marker of the myocardium’s ability to convert substrate into ATP for active processes, and a sensitive index of the energetic state of the myocardium [7]. In this study, in line with other CMR findings showing reassuringly normal assessments, we have not detected any significant abnormality in the cardiac PCr/ATP ratio in patients with long COVID-19 syndrome, suggesting preserved function of the myocardial metabolic machinery.

## Limitations

The small sample size and the cross-sectional nature of the study assessments are important limitations which prevent the generalizability of our findings and accuracy of prevalence estimates. Quality of life data was also only available in 16 patients, which may underestimate the severity of symptoms in this cohort. As such, this study should be considered preliminary and exploratory. The complexity of the imaging protocol and associated financial costs may limit its widespread use, but if feasible larger multicenter studies with extended follow-up will provide more definitive answers.

As short axis cine stack was performed after adenosine administration, this could have potentially masked minor differences in LV function between the 2 groups in theory [31]. However, this is unlikely to be significant, as all tissue characteristics and stress myocardial perfusion were normal in our cohort and therefore the impact of adenosine on LV function would likely be comparable. While healthy subjects reported no symptoms nor displayed any signs of an active infection, COVID-19 PCR testing was not performed to exclude an asymptomatic SARS-CoV-2 infection and neither was antibody testing performed to exclude a prior infection.

## Conclusions

In this single centre prospective study, we found that the overwhelming majority of patients with a clinical diagnosis of long COVID-19 syndrome with a mild acute phase of SARS-CoV-2 infection and no prior cardiovascular disease or comorbidities, exhibited no significant abnormalities in cardiac energetics, structure or function, myocardial blood flow or tissue characteristics. Larger/multicenter studies are, however, needed to evaluate the generalizability of these findings in a larger population and to better understand the pathophysiology of long COVID-19 syndrome.

## Data Availability

The datasets used and/or analysed during the current study are available from the corresponding author on reasonable request.

## Abbreviations

^31^P-CMRS: 31-Phosphorus cardiovascular magnetic resonance spectroscopy
AHA: American Heart Association
ATP: Adenosine triphosphate
BMI: Body mass index
BP: Blood pressure
bSSFP: Balanced steady state free precession
CAD: Coronary artery disease
CMR: Cardiovascular magnetic resonance imaging
COVID-19: Coronavirus Disease 2019
CSI: Chemical shift imaging
ECV: Extracellular volume fraction
eGFR: Estimated glomerular filtration rate
GLS: Global longitudinal strain
IQR: Interquartile range
LA: Left atrium/left atrial
LGE: Late gadolinium enhancement
LTHT: Leeds Teaching Hospital NHS Trust
LV: Left ventricle/left ventricular
LVEDV: Left ventricular end-diastolic volume
LVEF: Left ventricular ejection fraction
MBF: Myocardial blood flow
MOLLI: Modified look locker inversion recovery
MPRI: Myocardial perfusion index
NT-proBNP: N-terminal pro hormone b type natriuretic peptide
PCr/ATP: Phosphocreatine to ATP ratio
RV: Right ventricle/right ventricular
RVEF: Right ventricular ejection fraction
SARS-CoV-2: Severe Acute Respiratory Syndrome Coronavirus 2
